# Correlation Between Objective and Subjective High-Pitched Voice Impairment in Patients After Thyroid Surgery

**DOI:** 10.3389/fendo.2021.788878

**Published:** 2021-11-11

**Authors:** Tzu-Yen Huang, Wing-Hei Viola Yu, Feng-Yu Chiang, Che-Wei Wu, Shih-Chen Fu, An-Shun Tai, Yi-Chu Lin, Hsin-Yi Tseng, Ka-Wo Lee, Sheng-Hsuan Lin

**Affiliations:** ^1^ International Thyroid Surgery Center, Department of Otolaryngology-Head and Neck Surgery, Kaohsiung Medical University Hospital, Faculty of Medicine, College of Medicine, Kaohsiung Medical University, Kaohsiung, Taiwan; ^2^ Department of Biological Science and Technology, National Yang Ming Chiao Tung University, Hsinchu, Taiwan; ^3^ Department of Biological Science and Technology, National Chiao Tung University, Hsinchu, Taiwan; ^4^ Department of Otolaryngology-Head and Neck Surgery, E-Da Hospital, Kaohsiung, Taiwan; ^5^ School of Medicine, College of Medicine, I-Shou University, Kaohsiung, Taiwan; ^6^ Department of Otolaryngology-Head and Neck Surgery, Kaohsiung Municipal Siaogang Hospital, Kaohsiung Medical University Hospital, Faculty of Medicine, College of Medicine, Kaohsiung Medical University, Kaohsiung, Taiwan; ^7^ Institute of Statistics, National Yang Ming Chiao Tung University, Hsinchu, Taiwan; ^8^ Institute of Statistics, National Chiao Tung University, Hsinchu, Taiwan; ^9^ Department of Otolaryngology-Head and Neck Surgery, Kaohsiung Municipal Tatung Hospital, Kaohsiung Medical University Hospital, Faculty of Medicine, College of Medicine, Kaohsiung Medical University, Kaohsiung, Taiwan; ^10^ Institute of Data Science and Engineering, National Yang Ming Chiao Tung University, Hsinchu, Taiwan; ^11^ Institute of Data Science and Engineering, National Chiao Tung University, Hsinchu, Taiwan

**Keywords:** thyroid surgery, high-pitched voice impairment (HPVI), Index of Voice and Swallowing Handicap of Thyroidectomy (IVST), intraoperative neuromonitoring (IONM), voice stability

## Abstract

**Objectives:**

High-pitched voice impairment (HPVI) is not uncommon in patients without recurrent laryngeal nerve (RLN) or external branch of superior laryngeal nerve (EBSLN) injury after thyroidectomy. This study evaluated the correlation between subjective and objective HPVI in patients after thyroid surgery.

**Methods:**

This study analyzed 775 patients without preoperative subjective HPVI and underwent neuromonitored thyroidectomy with normal RLN/EBSLN function. Multi-dimensional voice program, voice range profile and Index of voice and swallowing handicap of thyroidectomy (IVST) were performed during the preoperative(I) period and the immediate(II), short-term(III) and long-term(IV) postoperative periods. The severity of objective HPVI was categorized into four groups according to the decrease in maximum frequency (Fmax): <20%, 20-40%, 40-60%, and >60%. Subjective HPVI was evaluated according to the patient’s answers on the IVST.

**Results:**

As the severity of objective HPVI increased, patients were significantly more to receive bilateral surgery (p=0.002) and have subjective HPVI (p<0.001), and there was no correlation with IVST scores. Among 211(27.2%) patients with subjective HPVI, patients were significantly more to receive bilateral surgery (p=0.003) and central neck dissection(p<0.001). These patients had very similar trends for Fmax, pitch range, and mean fundamental frequency as patients with 20-40% Fmax decrease (p>0.05) and had higher Jitter, Shimmer, and IVST scores than patients in any of the objective HPVI groups; subjective HPVI lasted until period-IV.

**Conclusion:**

The factors that affect a patient’s subjective HPVI are complex, and voice stability (Jitter and Shimmer) is no less important than the Fmax level. When patients have subjective HPVI without a significant Fmax decrease after thyroid surgery, abnormal voice stability should be considered and managed. Fmax and IVST scores should be interpreted comprehensively, and surgeons and speech-language pathologists should work together to identify patients with HPVI early and arrange speech therapy for them. Regarding the process of fibrosis formation, anti-adhesive material application and postoperative intervention for HPVI require more future research.

## Introduction

Thyroid surgery is a precise operation that requires, to the greatest extent possible, the preservation of the function of adjacent nerves when removing thyroid lesions. Intraoperative neural monitoring (IONM), as an adjunct technique for localizing and identifying the recurrent laryngeal nerve (RLN) and the external branch of the superior laryngeal nerve (EBSLN), has been widely studied and used in routine thyroid surgery (1, 2). The qualitative and quantitative information provided by IONM is far superior to visual identification of nerves alone, and standardized procedures and guidelines have been proposed in several studies, enabling more reliable recording of RLN status (3–7).

High-pitched voice impairment (HPVI) is not uncommon after thyroid surgery, and HPVI not only affects the performance of professional voice users but may cause a decline in the quality of life of nonprofessional voice users (8). The cricothyroid muscle (CTM) innervated by the EBSLN can lengthen the vocal fold and increase the fundamental frequency of vocal fold vibration to produce a higher-pitched voice; thus, many studies have focused on the relationship between the EBSLN and HPVI (9, 10). However, there are more studies that support the finding that HPVI can frequently appear in patients without RLN or EBSLN injury (11–13). After thyroidectomy, fibrosis may form between the strap muscles and laryngotracheal unit and impair the vertical movement (14), and may form in lateral extralaryngeal muscles when overtraction during the surgery. If fibrosis is formed between strap muscle and CTM, HPVI may be caused by limited movement of CTM.

To the best of our knowledge, there is currently no research investigating patients with objective and subjective HPVI after thyroid surgery by serial objective and subjective voice analysis. Furthermore, a standardized IONM procedure was applied in this study to exclude intraoperative RLN or EBSLN injury. This study aims to evaluate the correlation between objective and subjective HPVI in patients after thyroid surgery.

## Materials and Methods

**Figure 1:**
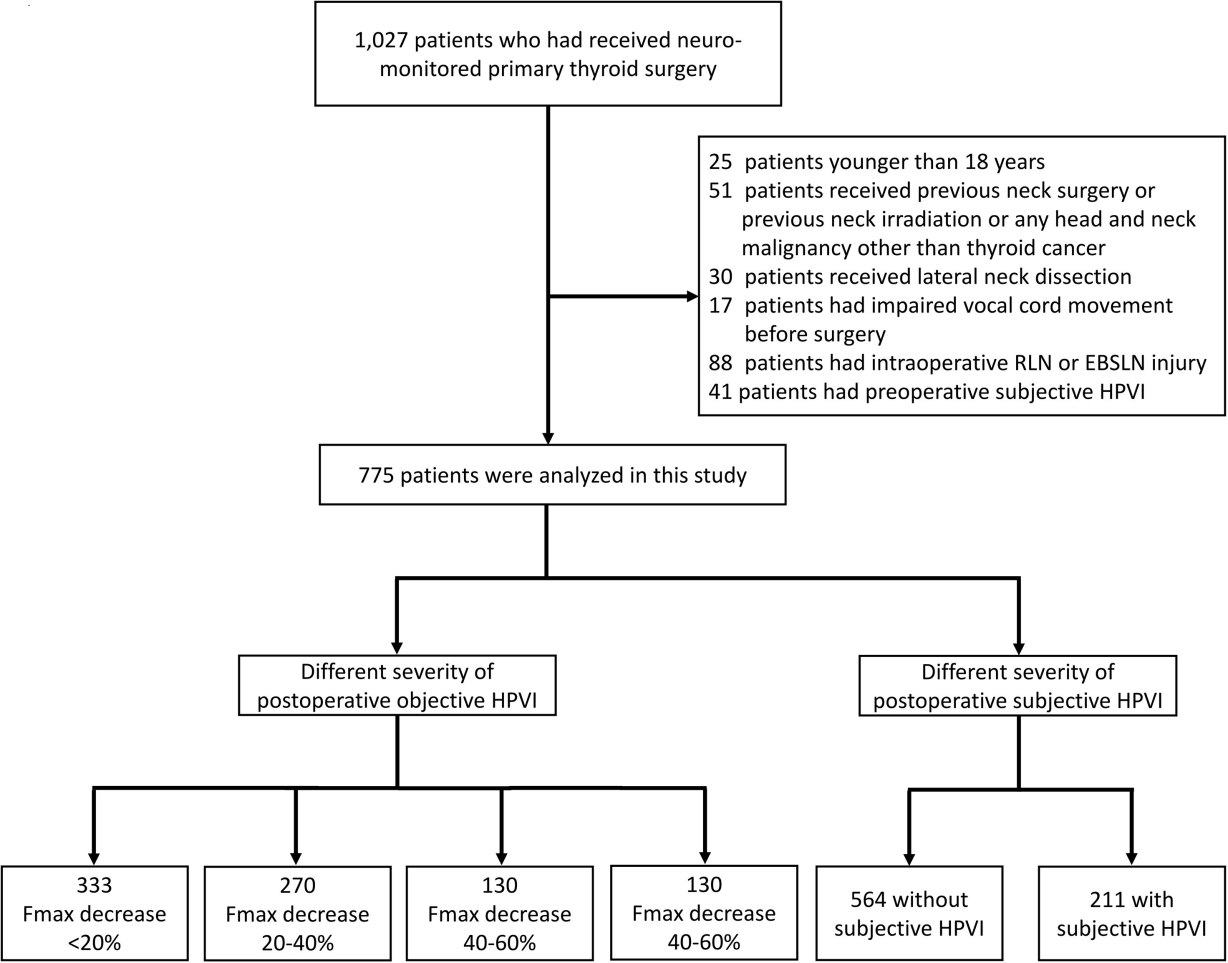
Flow diagram for inclusion and exclusion of patients. RLN, recurrent laryngeal nerve; EBSLN, external branch of superior laryngeal nerve; HPVI, high-pitched voice impairment; Fmax, maximum pitch frequency.

This study retrospectively enrolled 1,027 patients who underwent neuromonitored primary thyroid surgery at Kaohsiung Medical University Hospital from June 2013 to December 2019. The exclusion criteria included patients younger than 18 years at the time of surgery (n=25), patients who received previous neck surgery or previous neck irradiation, patients who had head and neck malignancy other than thyroid cancer (n=51), patients who underwent lateral neck dissection (n=30), patients who had impaired vocal fold motion before surgery (n=17), patients who had intraoperative RLN or EBSLN injury (n=88), and patients who had preoperative subjective HPVI (n=41). A flow diagram illustrating the inclusion and exclusion of patients is shown in **Figure 1**. 775 patients were analyzed in this study. All surgeries were performed by experienced thyroid surgeons (F-Y. C, C-W. W, and T-Y. H) in the IONM team at Kaohsiung Medical University Hospital (3, 15). Ethical approval for this study was obtained from the Kaohsiung Medical University Hospital Institutional Review Board (KMUHIRB-E(I)-20200358). In all patients, vagus nerve and RLN function were routinely evaluated using the standard (V1-R1-S1-S2-R2-V2) procedure under IONM (2, 3). The EMG amplitudes of R2 and R1 signals were compared, and an R2 signal showing a >50% decrease from the R1 signal was defined as RLN injury. EBSLN integrity is measured by EBSLN monitoring according to international guidance (5). The five steps included the following: E (Expose the space harboring the EBSLN), B (Bluntly dissect tissues), S (Stimulate tissues during dissection), L (Look for CTM twitch), and N (Navigate the dissection using the nerve mapping technique). S2 stimulation at the most proximal EBSLN was performed at the end of the operation. Once CTM twitching could not observed after S2 stimulation, it was defined as EBSLN injury.

Patient information, including gender, age, surgical extent (unilateral or bilateral surgery), pathology report (benign or malignant), and central neck dissection (CND), was recorded and compared between groups. Laryngofiberscopy was documented by video in all patients before surgery and 2 weeks after surgery, and no patient in this study had preoperative asymmetric vocal fold motion.

### Objective and Subjective Voice Analysis

Subjective and objective voice analyses were performed for all patients in four periods: preoperative period (period-I, within 2 months before surgery); immediate postoperative period (period-II, median duration of 3 days, range of 1-7 days); short-term postoperative period (period-III, median duration of 12 days, range of 7-30 days); and long-term postoperative period (period IV, median duration of 40 days, range of 30-90 days).

All objective voice analyses were performed by a single experienced speech-language pathologist (WHV. Y). The Multidimensional Voice Program (MDVP, model 5105, version 3.1.7; KayPENTAX, USA) results included mean fundamental frequency (Mean F0), Jitter, Shimmer and noise-to-harmonic ratio (NHR). The Voice Range Profile (VRP, model 4326, version 3.3.0; KayPENTAX, USA) results included maximum pitch frequency (Fmax), minimum pitch frequency (Fmin), and pitch range (PR). PR was defined as the number of semitones between Fmax and Fmin. The comparison of preoperative Fmax and the worst Fmax more than 7 days after surgery was used as the Fmax grouping. The groups were divided into four categories according to Fmax decrease: <20%, 20-40%, 40-60%, and >60%.

The subjective voice analysis was evaluated by the Index of Voice and Swallowing Handicap of Thyroidectomy (IVST) (**Table 1**). The IVST was designed based on the main symptoms observed before and after thyroid surgery. Each of the 10 questionnaire items in this subjective assessment is assigned a score of 0 (never), 1 (sometimes), or 2 (always). The voice domain (IVST-V) includes items 1-7 and has a score range of 0-14. The swallowing domain (IVST-S) includes items 8-10 and has a score range of 0 to 6. Thus, the total IVST score (IVST-T) has a score range of 0 to 20. The sixth question on the IVST is “I find it difficult to make a high-pitched voice.” All patients in this study answered “never” (0 points) to this question preoperatively. The patients who answered “sometimes” (1 point) or “always” (2 points) at least 7 days postoperatively were defined as having postoperative subjective HPVI.

**Table 1:** Index of voice and swallowing handicap of thyroidectomy (IVST).

Questions	Never (0 point)	Sometimes (1 point)	Always (2 points)
Voice domain			
1. My overall voice quality is abnormal.	0	1	2
2. My voice difficulties restrict personal and social life.	0	1	2
3. I feel my voice is hoarse.	0	1	2
4. I feel as though I have to strain to produce voice.	0	1	2
5. The sound of my voice varies throughout the day.	0	1	2
6. I find it difficult to make a high-pitched voice.	0	1	2
7. I find it difficult to make a low-pitched voice.	0	1	2
	**IVST-V = ______ (Range from 0-14)**		
Swallowing domain			
8. I feel strained when I speak or swallow.	0	1	2
9. I choke when I drink (water or tea).	0	1	2
10. I choke when I eat.	0	1	2
	**IVST-S = ______ (Range from 0-6)**		
Total score	**IVST-T = ______ (Range from 0-20) **		

The equation for calculating postoperative change in objective voice analysis data was Δ= (B - A)/A; the equation for calculating postoperative change in subjective voice analysis data was Δ= B – A, where A and B are the preoperative and postoperative values, respectively.

### Statistical Analysis

To analyze the variables, independent t tests, Pearson chi-square tests, and ANOVA tests were performed using R software (version-3.4). A two-tailed p value less than 0.05 was considered statistically significant.

## Results

### Demographic Characteristics of Patients With Different Severity of Postoperative Objective HPVI

775 patients were analyzed in this study. The comparison between different severity of Fmax decrease (<20%, 20-40%, 40-60%, and >60%) is shown in **Table 2**. Age, gender and pathology report showed no significant difference between groups. Significantly more bilateral surgeries were performed in the group with higher severity of Fmax decrease (p=0.002). There were significant differences (p<0.001) in the proportion of patients receiving CNDs among the different Fmax decrease groups but not in the order of Fmax decrease severity. Significantly more patients with higher severity of Fmax decrease had subjective HPVI (p<0.001).

**Table 2:** Demographic characteristics of patients with different severity of objective high-pitched voice impairment (HPVI).

Total 775 patients	Fmax decrease				p value
	<20%	20-40%	40-60%	>60%	
Case number	333 (42.3%)	270 (34.8%)	130 (16.8%)	42 (5.4%)	
Age ± SD	50.9 ± 12.8	51.8 ± 13.1	53.4 ± 12.8	55.6 ± 13.3	0.073
Gender (%)					0.139
male	48 (14.4%)	50 (18.5%)	27 (20.8%)	6 (14.3%)
female	285 (85.6%)	220 (81.5%)	103 (79.2%)	36 (85.7%)
Surgical extent (%)					0.002
unilateral	105 (31.5%)	70 (25.9%)	26 (20.0%)	5 (11.9%)
bilateral	228 (68.5%)	200 (74.1%)	104 (80.0%)	37 (88.1%)
CND					<0.001
without	294 (88.3%)	231 (85.6%)	101 (77.7%)	36 (85.7%)
with	39 (11.7%)	39 (14.4%)	29 (22.3%)	6 (14.3%)
Pathology					0.547
benign	228 (68.5%)	183 (67.8%)	80 (61.5%)	30 (71.4%)
malignant	105 (31.5%)	87 (32.2%)	50 (38.5%)	12 (28.6%)
Patients with subjective HPVI	29 (8.7%)	74 (27.4%)	73 (56.2%)	35 (83.3%)	<0.001

SD, standard deviation; CND, Central neck dissection.

### Demographic Characteristics of Patients With and Without Postoperative Subjective HPVI

The comparison is shown in **Table 3**. Age and pathology report showed no significant differences between groups. The proportion of female patients was significantly higher in patients with subjective HPVI than in those without subjective HPVI (88.2% vs. 81.2%, p=0.022). The proportion of patients who received bilateral surgery was significantly higher in patients with subjective HPVI than in those without subjective HPVI (81.0% vs. 70.6%, p=0.003). The proportion of patients who received CND was significantly higher in patients with subjective HPVI than in patients without subjective HPVI (25.1% vs. 10.6%, p<0.001).

**Table 3:** Demographic characteristics of patients with and without subjective high-pitched voice impairment (HPVI).

Total 775 patients	Without subjective HPVI	With subjective HPVI	p value
Case number	564 (72.8%)	211 (27.2%)	
Age ± SD	51.5 ± 12.7	52.9 ± 11.6	0.163
Gender (%)			0.022
male	106 (18.8%)	25 (11.8%)	
female	458 (81.2%)	186 (88.2%)	
Surgical extent (%)			0.003
unilateral	166 (29.4%)	40 (19.0%)	
bilateral	398 (70.6%)	171 (81.0%)	
CND			<0.001
without	504 (89.4%)	158 (74.9%)	
with	60 (10.6%)	53 (25.1%)	
Pathology			0.315
benign	385 (68.3%)	136 (64.5%)	
malignant	179 (31.7%)	75 (35.5%)	

SD, standard deviation; CND, central neck dissection.

### Voice Parameter Changes in Patients With Different Severity of Objective HPVI And Patients With Subjective HPVI

**Figure 2:**
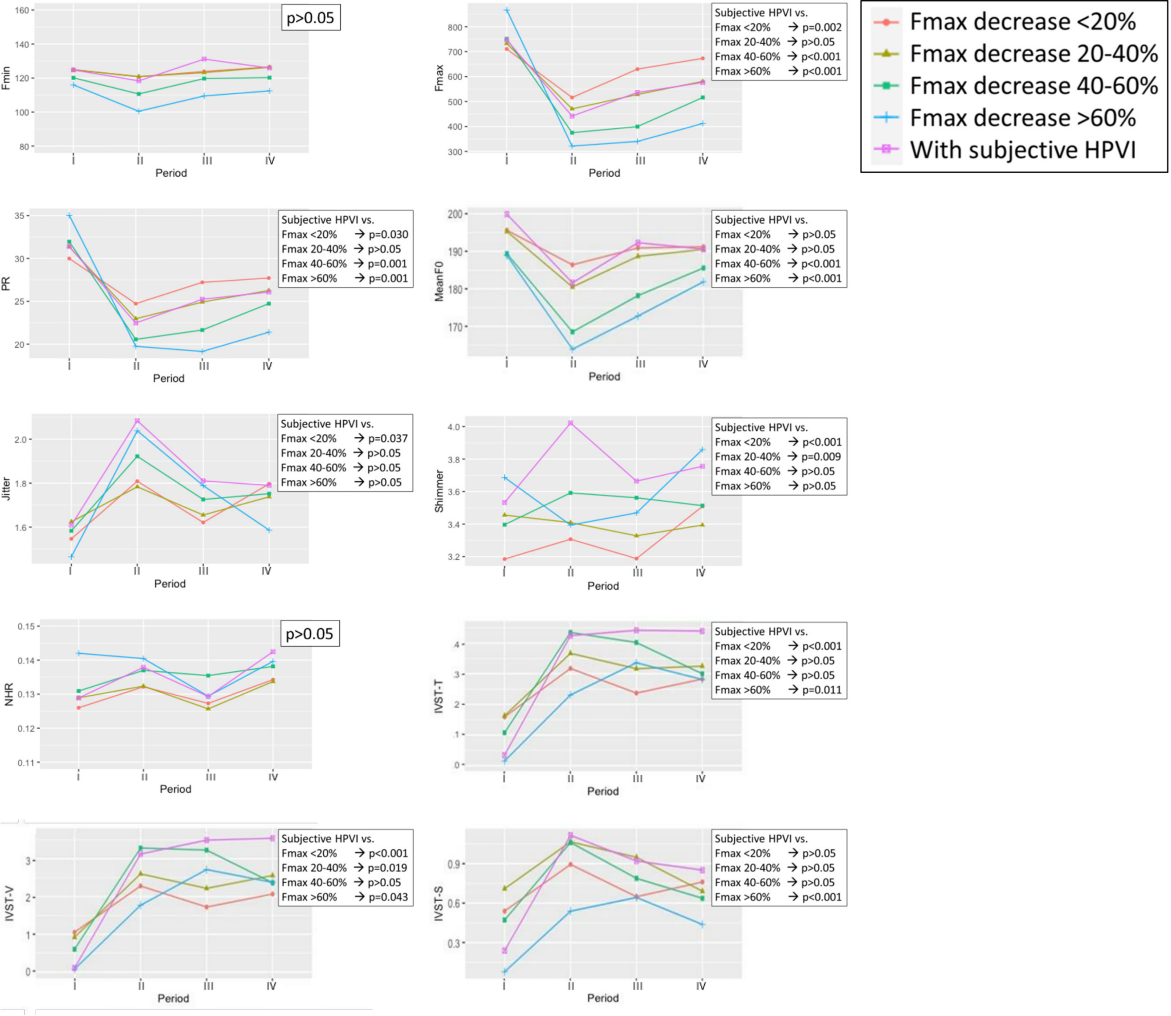
Voice parameter changes in patients with different severity of objective high-pitched voice impairment (HPVI) and patients with subjective HPVI in each follow-up period. Red line = Fmax decrease <20%; Olive line = Fmax decrease 20-40%; Green line = Fmax decrease 40-60%; Blue line = Fmax decrease > 60%; Purple line = Patients with subjective HPVI. period-I = Preoperative period (within 2 months before surgery); period-II = Immediate postoperative period (median duration of 3 days; range of 1-7 days); period-III = Short-term postoperative period (median duration of 12 days; range of 7-30 days); period-IV = Long-term postoperative period (median duration of 40 days, range of 30-90 days). A p value less than 0.05 was considered statistically significant.

The voice parameter changes are shown in **Figure 2**. There were no significant differences between groups for Fmin or NHR. The Fmax and PR of patients with subjective HPVI were not significantly different than those of patients with an Fmax 20-40% decrease, and their results in the chart show a high degree of overlap. There was a significant difference in Mean F0 for patients with subjective HPVI and patients with Fmax decreases of 40-60% and >60%. The greater the Fmax decrease was, the lower the Mean F0.

The greater the Fmax decrease was, the higher the Jitter and Shimmer values. The Jitter and Shimmer values for patients with subjective HPVI were higher than those for patients in any of the objective HPVI groups.

The IVST-T, IVST-V and IVST-S scores showed no correlation with the different degrees of Fmax decrease. The postoperative IVST-T and IVST-V values for patients with subjective HPVI were higher than those for patients in any of the objective HPVI groups, especially during period IV.

## Discussion

In this study, we investigated the correlation between subjective and objective HPVI in patients after monitored thyroid surgery and confirmed normal RLN/EBSLN function. We found that as the Fmax severity increased, significantly more patients received bilateral surgery (p=0.002) and had subjective HPVI (p<0.001) (**Table 2**). The objective HPVI patients tended to have decreased PR and Mean F0 and increased Jitter and Shimmer. However, the severity of objective HPVI was not correlated with subjective IVST scores (**Figure 2**). When patients had subjective HPVI, significantly more of them received bilateral surgery (p=0.003) and CND (p<0.001) (**Table 3**). Patients with subjective HPVI had very similar trends for Fmax, PR and Mean F0 as patients with an Fmax decrease of 20-40%. In addition, patients with subjective HPVI had higher Jitter, Shimmer, IVST-T and IVST-V levels than patients in any of the objective HPVI groups, and subjective HPVI lasted until period-IV (**Figure 2**). These data show the factors that affect a patient’s subjective HPVI are very complex and voice stability (Jitter and Shimmer) is no less important than Fmax level. Therefore, when patients have subjective HPVI without a significant Fmax decrease after thyroid surgery, abnormal voice stability should be considered and managed.

In this study, bilateral surgery and CND were factors that were related to subjective HPVI in patients, while only bilateral surgery was a factor for Fmax decrease. The voice parameters, including PR and Mean F0, were correlated with Fmax decrease. CTM contraction elongates the vocal ligaments; when the tension of the vocal ligament is insufficient, Fmax, PR and Mean F0 will decrease (9, 16, 17). In this study, all patients received IONM-assisted thyroidectomy, all EBSLNs were stimulated, and CTM twitches were observed during surgery. There are fewer neurological factors for decreased CTM function, and patients with decreased CTM function are more likely to have decreased muscle contraction. During the operation, the fascia of CTM is usually left intact, but when there is local adhesion or the tumor is adjacent, it may still be partially exposed. After thyroid tumor resection, the strap muscles divided after the midline approach are routinely sutured; however, fibrosis formation between the CTM and strap muscles is still inevitable. Therefore, future studies are needed to verify whether anti-adhesive interventions can improve fibrosis on CTM, and voice parameters, including Fmax, PR and Mean F0, may be applicable in outcome evaluation.

The factors that affect subjective HPVI are much more complicated, and the subjective and objective voice parameters showed less correlation. Patients with a greater Fmax decrease had a higher proportion of subjective HPVI, however, the trends for Fmax, PR and Mean F0 in subjective HPVI patients were very similar as patients with an Fmax decrease of 20-40%, rather than closer to the more severe objective HPVI groups. This indicated that Fmax was not the only factor that causes subjective HPVI. For example, a higher proportion of CND in patients with subjective HPVI may imply that even if the dissection is performed away from CTM, there are still other factors that cause subjective HPVI. Although a higher degree of Fmax decrease was associated with higher values for Jitter and Shimmer, postoperative Jitter and Shimmer values were higher for patients with subjective HPVI than for patients in any of the objective HPVI groups. The factors related to subjective HPVI may be highly correlated with voice stability rather than simple CTM contractility. All patients enrolled in this study had comprehensive EMG signal recording, and none of the patients had EMG amplitude decreases >50%. The possible factors associated with decreased voice stability during thyroid surgery include fibrosis of the strap muscle or other extrinsic muscles or local soft tissue fibrosis (18). Therefore, varied anti-adhesive materials applied in thyroidectomy with different surgical routes may have a role in preventing fibrosis-related symptoms (19).

IVST-T and IVST-V showed no correlation with Fmax decrease, and patients with subjective HPVI had greater IVST-T and IVST-V, especially during period-IV. The factors most affecting subjective voice in the immediate postoperative and short-term postoperative period include intubation, laryngeal edema, and wound factors that restrict patients’ phonation (20). However, during period-IV, the influence of these factors gradually subsided, suggesting that postoperative fibrosis may play an important role and may mainly affect voice stability. To prevent fibrosis after thyroidectomy, instructing patients to perform stretching exercises to reduce the symptoms of postoperative neck discomfort is suggested (21). To determine whether postoperative fibrosis is related to CTM, the Fmax and IVST scores should be interpreted comprehensively. Surgeons and speech-language pathologists should work together to identify patients with postoperative HPVI early and arrange speech therapy for them. For example, when patients have persistent subjective HPVI 3 months after thyroid surgery and the objective voice parameters have gradually improved, the treatment target should include strap muscles and other extrinsic muscles not limited to the CTM. The precise duration of fibrosis-related HPVI remains unclear, and the treatment strategy also needs to be further studied.

Unlike objective voice analysis, subjective voice analysis based on data collected *via* a questionnaire will inevitably exhibit response bias (22). Among the patients with subjective HPVI, there were significantly more female patients. Females may be more likely to notice a change to a higher voice pitch, and males may be more likely to ignore it; a similar finding was also described by Park et al. (12) Acquiescence bias is a category of response bias in which respondents have a tendency to choose a positive response option (23), and approximately 10% to 20% of respondents exhibit this behavior (24). In our study, the IVST-S of the subjective HPVI patients had a higher value than that for any of the objective HPVI groups. Although the difference is not significant, it is still hard to identify an anatomical explanation. Given that the subjective HPVI patients had higher IVST-V values, there may have been an acquiescence bias that increased the average IVST-S value. It is notable that the IVST-S of subjective HPVI patients decreased during period IV, similar to the trend observed for the IVST-S of objective HPVI patients; this was not observed for IVST-T and IVST-V. Fibrosis as a main factor influencing HPVI has only a limited effect on swallowing. Short-term swallowing impairment and long-term improvements were also described in the study by Lombardi et al. (14)

This study has several limitations. First, patients with preoperative subjective HPVI were excluded from this study, as the purpose was to exclude other long-term nonsurgical factors causing HPVI. Patients with preoperative HPVI may need preoperative preventive treatment and postoperative evaluation and management, and this requires further research and analysis. Second, patients with thyroid cancer may require lateral neck dissection, and how surgical dissection and postoperative fibrosis impact HPVI remains unclear and requires future research. Last, Jitter and Shimmer of MDVP is measured by the fundamental frequency of patients’ voices. Evaluating Jitter and Shimmer with a high-pitched frequency (high-pitched MDVP) or other novel objective voice analysis parameters could provide more information about high-pitched voice stability.

## Conclusion

In this study, the demographic characteristics and voice parameter changes of patients with subjective and objective HPVI after thyroidectomy were evaluated. A decrease in Fmax accompanied by PR and a decrease in Mean F0 showed an association with CTM contraction reduction. The factors that cause subjective HPVI in thyroidectomy patients are very complex, and voice stability (Jitter and Shimmer) is no less important than Fmax level. When patients have subjective HPVI without a significant Fmax decrease after thyroid surgery, abnormal voice stability should be considered and managed. Fmax and IVST scores should be interpreted comprehensively, and surgeons and speech-language pathologists should work together to identify patients with HPVI early and arrange speech therapy for them. Regarding the process of fibrosis formation, anti-adhesive material application and postoperative intervention for HPVI require more research in the future.

## Data Availability Statement

The original contributions presented in the study are included in the article. Further inquiries can be directed to the corresponding author.

## Ethics Statement

The studies involving human participants were reviewed and approved by the Kaohsiung Medical University Hospital Institutional Review Board (KMUHIRB-E(I)-20200358). The ethics committee waived the requirement of written informed consent for participation.

## Author Contributions

Supervision – F-YC, C-WW, K-WL, and S-HL. Materials – T-YH, W-HV, F-YC, and C-WW. Data collection and processing – T-YH, W-HV, and S-HL. Analysis and interpretation- T-YH, S-CF, A-ST, and S-HL. Literature search - T-YH, W-HV, Y-CL, H-YT, and S-HL. Writing manuscript – All authors. All authors have read and agreed to the published version of the manuscript.

## Funding

This study was supported by grants from Kaohsiung Medical University Hospital, Kaohsiung Medical University (KMUH109-9M44), Kaohsiung Municipal Siaogang Hospital/Kaohsiung Medical University Research Center grants (KMHK-DK(C)110009, I-109-04, H-109-05, I-108-02), and Ministry of Science and Technology (MOST 108-2628-B-037-006, MOST 109-2628-B-037-014, MOST 110-2636-B-009-008, MOST 110-2314-B-037-104-MY2, MOST 110-2314-B-037-120), Taiwan.

## Conflict of Interest

The authors declare that the research was conducted in the absence of any commercial or financial relationships that could be construed as a potential conflict of interest.

## Publisher’s Note

All claims expressed in this article are solely those of the authors and do not necessarily represent those of their affiliated organizations, or those of the publisher, the editors and the reviewers. Any product that may be evaluated in this article, or claim that may be made by its manufacturer, is not guaranteed or endorsed by the publisher.
